# Autophagy in the Central Nervous System and Effects of Chloroquine in Mucopolysaccharidosis Type II Mice

**DOI:** 10.3390/ijms20235829

**Published:** 2019-11-20

**Authors:** Mitsuyo Maeda, Toshiyuki Seto, Chiho Kadono, Hideto Morimoto, Sachiho Kida, Mitsuo Suga, Motohiro Nakamura, Yosky Kataoka, Takashi Hamazaki, Haruo Shintaku

**Affiliations:** 1 Multi-Modal Microstructure Analysis Unit, RIKEN-JEOL Collaboration Center, Hyogo 650-0047, Japan; msuga@jeol.co.jp (M.S.); monakamu@jeol.co.jp (M.N.); kataokay@riken.jp (Y.K.); 2 Laboratory for Cellular Function Imaging, RIKEN Center for Biosystems Dynamics Research, Hyogo 650-0047, Japan; 3 Department of Medical Genetics, Osaka City University Graduate School of Medicine, Osaka 545-8585, Japan; 4 Department of Pediatrics, Osaka City University Graduate School of Medicine, Osaka 545-8585, Japan; kadono@med.osaka-cu.ac.jp (C.K.); hammer@med.osaka-cu.ac.jp (T.H.); shintakuh@med.osaka-cu.ac.jp (H.S.); 5 JCR Pharmaceuticals Co., Ltd., Hyogo 659-0021, Japan; morimoto-h@jcrpharm.co.jp (H.M.); kida-s@jcrpharm.co.jp (S.K.); 6 Japan Electron Optics Laboratory (JEOL) Ltd., Tokyo 196-8558, Japan

**Keywords:** autophagy, brain, chloroquine, intellectual disability, mucopolysaccharidosis, neuron

## Abstract

Mucopolysaccharidosis type II (MPS II) is a rare lysosomal storage disease (LSD) involving a genetic error in iduronic acid-2-sulfatase (IDS) metabolism that leads to accumulation of glycosaminoglycans within intracellular lysosomes. The primary treatment for MPS II, enzyme replacement therapy, is not effective for central nervous system (CNS) symptoms, such as intellectual disability, because the drugs do not cross the blood–brain barrier. Recently, autophagy has been associated with LSDs. In this study, we examined the morphologic relationship between neuronal damage and autophagy in IDS knockout mice using antibodies against subunit c of mitochondrial adenosine triphosphate (ATP) synthetase and p62. Immunohistological changes suggesting autophagy, such as vacuolation, were observed in neurons, microglia, and pericytes throughout the CNS, and the numbers increased over postnatal development. Oral administration of chloroquine, which inhibits autophagy, did not suppress damage to microglia and pericytes, but greatly reduced neuronal vacuolation and eliminated neuronal cells with abnormal inclusions. Thus, decreasing autophagy appears to prevent neuronal degeneration. These results suggest that an autophagy modulator could be used in addition to conventional enzyme replacement therapy to preserve the CNS in patients with MPS II.

## 1. Introduction

Mucopolysaccharidosis type II (MPS II)—also known as Hunter’s syndrome—is a type of lysosomal storage disease (LSD) [1,2,3]. MPS II is an X-linked recessive disease caused by a congenital defect of iduronate-2-sulfatase that induces the accumulation of glycosaminoglycans in intracellular lysosomes. Because lysosomal enzymes are housekeeping proteins, LSDs—including MPS II—cause systemic signs, such as respiratory infection, hepatosplenomegaly, characteristic facial features, bone deformities, corneal opacity, joint contraction, and intellectual disability (ID). In Japan, MPS II accounts for approximately 60% of all mucopolysaccharidoses, 70% of which are severe forms associated with severe ID [4]. The severe neurological signs of MPS II start with developmental delay in early childhood and gradually progress from mild to severe ID and a bedridden state in the late stage. At present, both mild and severe MPS II are treated with enzyme replacement therapy (ERT). ERT can improve physical manifestations such as hepatosplenomegaly and is effective for improving skin softness, joint movement, and lung and respiratory functions; it also reduces the urinary excretion of glycosaminoglycans [5,6]. The neuronal impairment is irreversible, however, ERT has no effect on ID and other neuronal manifestations at all [7], because the intravenously administered enzyme cannot cross the blood–brain barrier. Elucidating the pathology of the brain involvement in MPS II and developing therapies focused on improving the central nervous system (CNS) manifestations are important issues for MPS II patients, as well as for medical economics and society. It has recently been suggested that a disturbance of lysosomal enzymes disrupts basic cell homeostasis, which is involved in autophagy, exocytosis, and the cell-membrane repair mechanism, resulting in the cell death associated with LSD [8,9,10]. Both suppression and enhancement of autophagy have been reported to be promising treatments for LSD. Inactivation of *Atg7*, which is involved in autophagy, attenuated signs in a mouse model of Pompe disease [11]. Conversely, overexpression of the autophagy-stimulating gene transcription factor EB also suppressed signs in a mouse model of Pompe disease [12]. Furthermore, abnormal activation of autophagy has been reported in a mouse model of GM1 gangliosidosis, which is characterized mainly by neurodegenerative signs [13]. Autoregulation (acceleration or suppression) of autophagy leads to signs of LSDs, and many available drugs that target autophagy have been described [8,9,14]. The progression of neurodegenerative diseases such as Alzheimer’s disease [15,16] and Huntington’s disease is suppressed by the acceleration of autophagy. Catabolic dysfunction and destabilization of lysosomes are common mechanisms of neurodegenerative diseases such as beta-propeller protein-associated neurodegeneration (also known as static encephalopathy of childhood with neurodegeneration in adulthood), which is caused by mutations in *WDR45* [17]. Autophagy is considered to be highly involved in the neuropathy of LSD. In 1997, Elleder et al. recognized that immunostaining of subunit c of mitochondrial ATP synthetase (SCMAS) was positive in the neurons of patients with neuronal ceroid lipofuscinosis (NCL), which is an LSD. In addition, they showed the accumulation of SCMAS in the brain neurons of patients with MPS I, II, and IIIA; Niemann–Pick disease A and C; and GM1 and GM2 gangliosidoses, and they suggested that the accumulation of SCMAS may be a common mechanism of neuropathy in LSDs [18]. Another marker of autophagy, p62, localizes to protein aggregates and abnormal mitochondria via a ubiquitin signal and is degraded by autophagy along with these structures [19]. To investigate the role of autophagy in the mechanisms of brain damage, we performed SCMAS and p62 antibody staining in iduronic acid-2-sulfatase (IDS) knockout (KO) mice, a model of MPS II. As in humans, the IDS gene in mice is located on the X chromosome, and X-linked recessive traits are seen in IDS-KO mice; 50% of males that are homozygous for the KO gene develop the disease. At three to four months after birth, bone deformity, joint disorders, and hepatomegaly are observed; neurologic signs appear; and movement becomes sluggish; the mice die 12 to 14 months after birth. We also examined the possible therapeutic effects of chloroquine as a treatment for neurodegeneration in our mouse model. We showed enhanced levels of p62 and SCMAS, suggestive of autophagy, as a result of enhanced initiation of autophagy, and/or reduced elimination of autophagic material in the neurons of the MPS II model mouse brain, and that administration of chloroquine was effective for treating neuronal degeneration.

## 2. Results

### 2.1. Vacuoles in Neurons, Microglia, and Pericytes in the CNS of IDS-KO Mice

**Figure 1:**
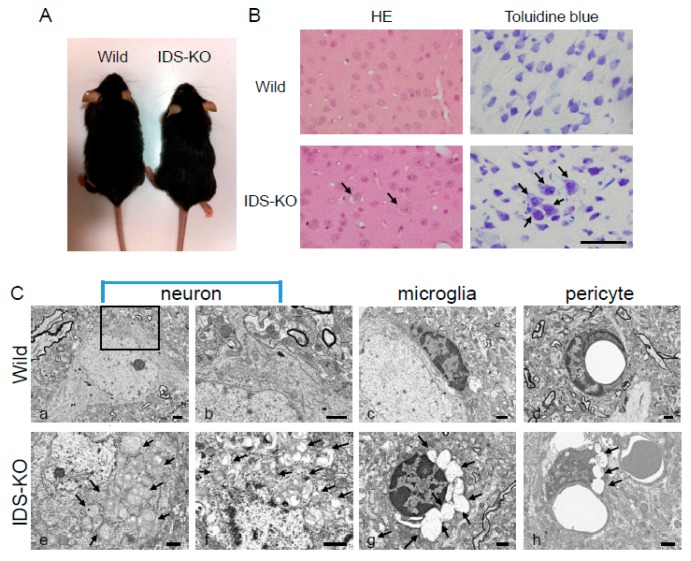
The appearance of wild-type and iduronic acid-2-sulfatase (IDS)-knockout (KO) mice. (**A**) The appearance of IDS-KO mice was not markedly changed. (**B**) Hematoxylin and eosin (HE) and toluidine blue (TB) staining of cerebral cortex from six-month-old mice. The neurons of the IDS-KO mice contained numerous vacuoles (arrows). Bar = 100 μm. (**C**) Electron micrographs of the cerebral cortex of wild-type (**a**–**d**) and IDS-KO (**e**–**h**) mice: (**a**,**b**,**e**,**f**) show neurons; (**c**,**g**) show microglia; (**d**,**h**) show pericytes. Panel b shows a higher magnification of the boxed area in (**a**). Numerous spiral structures and layered structures (**e**) and autophagy-like vacuoles (**f**) were found in the cytoplasm of the neurons in IDS-KO mice, and numerous vacuoles were observed in the cytoplasm of the microglia (**g**) and pericytes (**h**). Arrows indicate vacuoles. Bar = 1 μm.

The appearance of IDS-KO mice was not markedly different from that of the wild-type mice (Figure 1A), and there was no difference in weight between the two genotypes. Hematoxylin and eosin (HE) and toluidine blue (TB) staining of the cerebral cortex of IDS-KO mice revealed neurons and glial cells with small vacuoles in the cytoplasm (Figure 1B). Electron microscopy revealed a layered structure, with spiral inclusion bodies resembling “zebra bodies” [20] and large vacuoles in the cytoplasm of neurons of the cerebral cortex. Similar vacuoles were found in the microglia and pericytes of the blood vessel walls (Figure 1C e–h). However, these abnormalities were not observed in the cerebral cortex of wild-type mice (Figure 1C a–d).

### 2.2. Autophagy-Like Changes in the Cerebral Cortex of IDS-KO Mice

**Figure 2:**
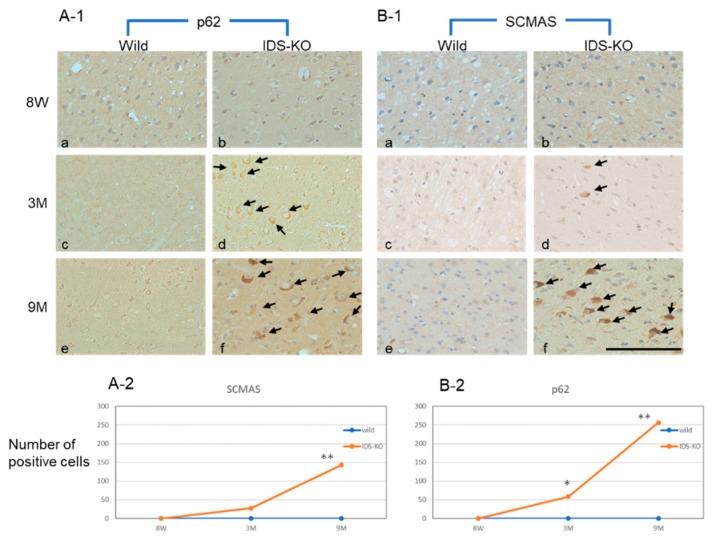
Immunostaining of P62 and subunit c of mitochondrial ATP synthetase (SCMAS) in the cerebral cortex. Immunostaining of P62 (**A-1**) and SCMAS (**B-1**) of specimens from wild-type (**a**,**c**,**e**) and IDS-KO (**b**,**d**,**f**) mice. Eight-week-old (8W) mice (**a**,**b**), three-month-old (3M) mice (**c**,**d**), nine-month-old (9M) mice (**e**,**f**). Arrows in A-1 and B-1 indicate p62-immunopositive cells and SCMAS-immunopositive neurons, respectively. Changes in the number of p62-positive cells (**A-2**) and SCMAS-positive cells (**B-2**) within 0.3 mm^2^ of cortex over time. Bar = 100 μm. * *p* < 0.05, ** *p* < 0.01, Welch’s *t*-test.

**Figure 3:**
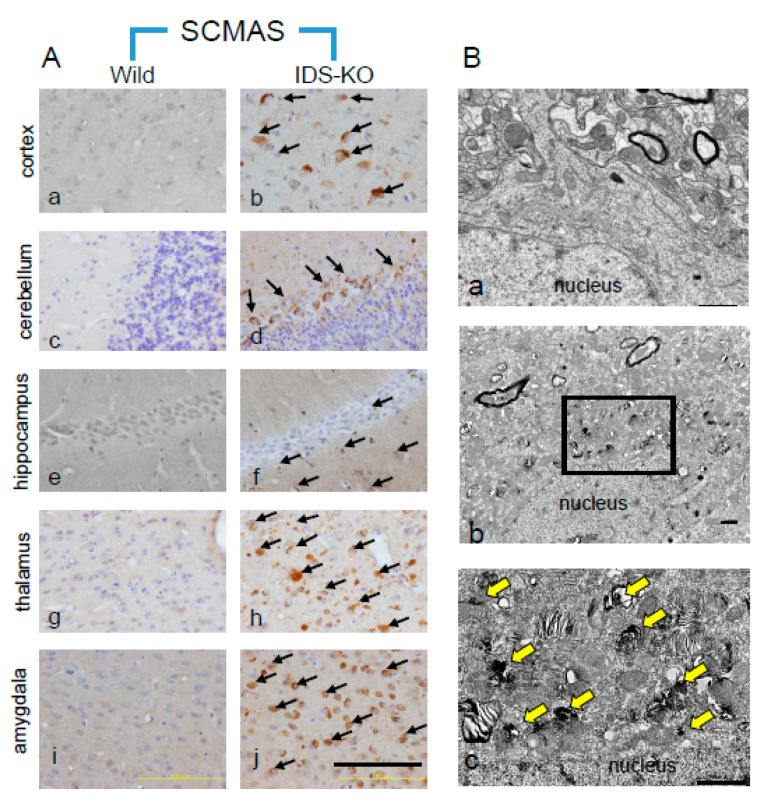
(**A**) Localization of SCMAS in the central nervous system (CNS) of wild-type and IDS-KO mice. No immunopositive cells were found in wild-type mice (**a**,**c**,**e**,**g**,**i**). In IDS-KO mice (**b**,**d**,**f**,**h**,**j**), immunopositive cells (arrows) were present in the cortex (**b**), cerebellum (**d**) hippocampus (**f**), thalamus (**h**), and amygdala (**j**). Bar = 100 μm. (**B**) SCMAS immunoelectron microscopy of the cerebral cortical neurons of wild-type (**a**) and IDS-KO mice (**b**,**c**). The immune response was negative in the wild-type mouse (**a**). SCMAS immunopositivity was observed at the vacuoles with single membranes (arrows) and in mitochondria that were partially swollen (**b**). Immunopositive reactions were observed in layered finger-print-like arrays and multilayered array structures ((**c**), arrows). Panel c shows the magnified view of an area indicated by a black rectangle in Panel b. Bar = 1 μm.

Anti-p62 and anti-SCMAS antibodies were used to examine the locations and timing of autophagy in the brain tissues of IDS-KO and wild-type mice. Neurons and glial cells in the IDS-KO mice were positive for p62 from three months of age, and p62 expression had increased at nine months, whereas in the wild-type mice, almost no positive cells were observed at any time (Figure 2 A-1,A-2). SCMAS-immunopositive cells also appeared at three months, and the numbers had increased at nine months (Figure 2B-1,B-2). SCMAS-immunopositive cells were found in each part of the IDS-KO brain examined at six months of age, including the cerebral cortex, cerebellum, hippocampus, thalamus, and amygdala, but no positive reaction was observed in any brain region of the wild-type mice (Figure 3A a–j).

### 2.3. Autophagy-Like Changes in Neurons, Microglia, and Pericytes of IDS-KO Mice

**Figure 4:**
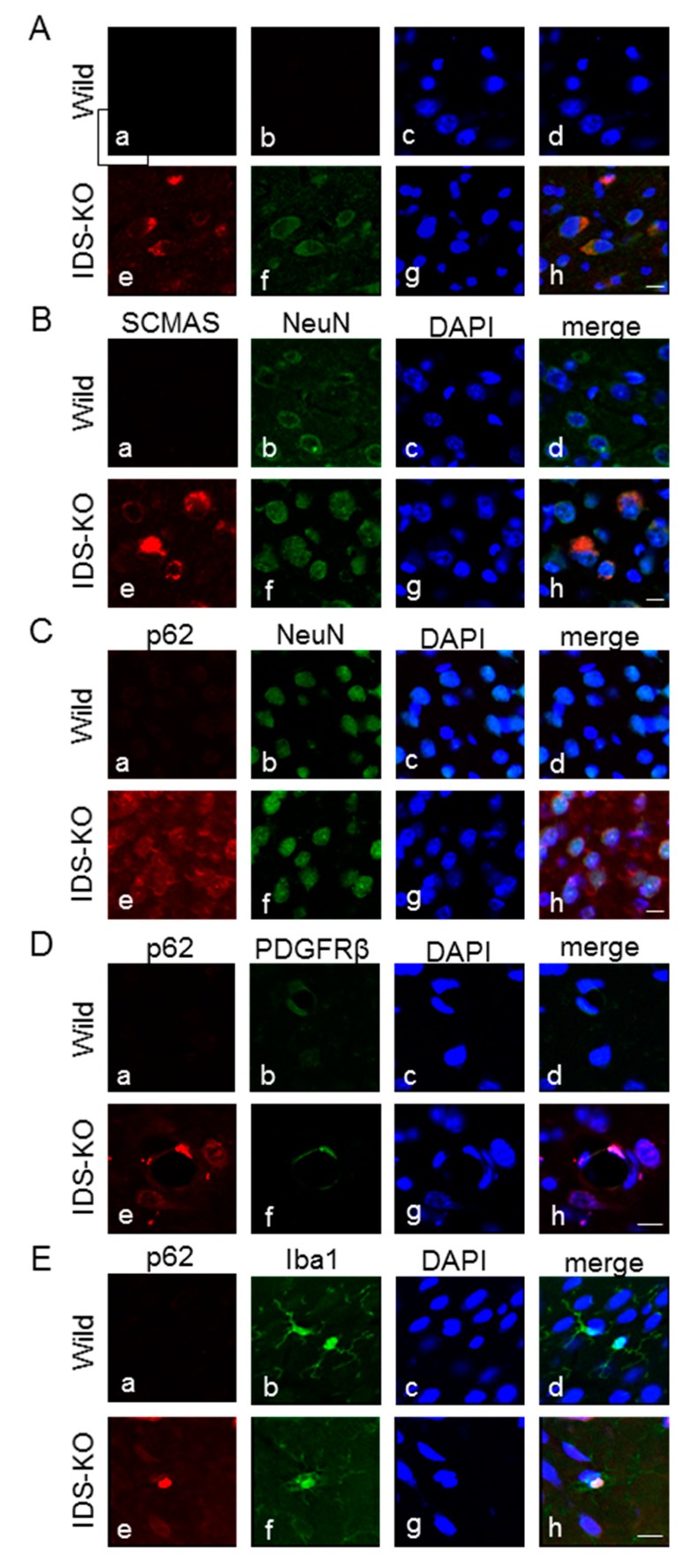
Double immunofluorescence staining with the indicated pairs of antibodies. Nuclei were counterstained with DAPI (blue). p62 (green) and SCMAS (red) immunopositivities were observed in the same cells of IDS-KO mice, confirming SCMAS as a marker of autophagy (**A**: **e**–**h**). Double immunofluorescence staining of SCMAS and NeuN (a neuronal marker) or of p62 and NeuN showed positive responses in the same cells (**B**,**C**: **e**–**h**). Double immunofluorescence staining of p62 and platelet-derived growth factor beta receptor (PDGFR-β) (a pericyte marker) showed positive responses in the same cells (**D**: **e**–**h**). Double immunofluorescence staining of p62 and iba 1 (a microglial marker) showed positive reactions for both in the same cells (**E**: **e**–**h**). Specimens from wild-type mice were immunonegative for p62 and SCMAS (**A**–**E**: **a**–**d**). Bar = 1 μm.

Subcellular localization of SCMAS in neurons was examined using immunoelectron microscopy with an SCMAS antibody. Normal-sized mitochondria were immunonegative, but hypertrophic mitochondria were immunopositive. The granules in large, single-membraned vacuoles in hypertrophic mitochondria were immunopositive for SCMAS (Figure 3B a–c). Also, lamellar or fingerprint-like structures in the cytoplasm were immunoreactive for SCMAS. That is, SCMAS immunoreactivity in neurons was found in vacuoles or fingerprint-like structures derived from mitochondria. Neurons without vacuoles were immunonegative. Subsequently, double immunofluorescence staining was performed to identify the cells immunopositive for p62 or SCMAS. The same cells were found to be immunopositive for SCMAS and p62, confirming that SCMAS-positive cells are also autophagy-positive (Figure 4A a–h). Double immunofluorescence staining with a combination of SCMAS and NeuN (a neuronal marker) or with p62 and NeuN also revealed double-positive cells, confirming the presence of autophagy in neurons in IDS-KO mice at six months of age (Figure 4B,C a–h). Double immunofluorescence staining with p62 and platelet-derived growth factor beta receptor (PDGFR-β) (a pericyte marker) confirmed autophagy of pericytes (Figure 4D a–h). Similarly, double immunofluorescence staining of p62 and iba1 (a microglial marker) confirmed autophagy of microglia (Figure 4E a–h).

### 2.4. Increase in Autophagy-Related Proteins in the Cerebral Cortex

**Figure 5:**
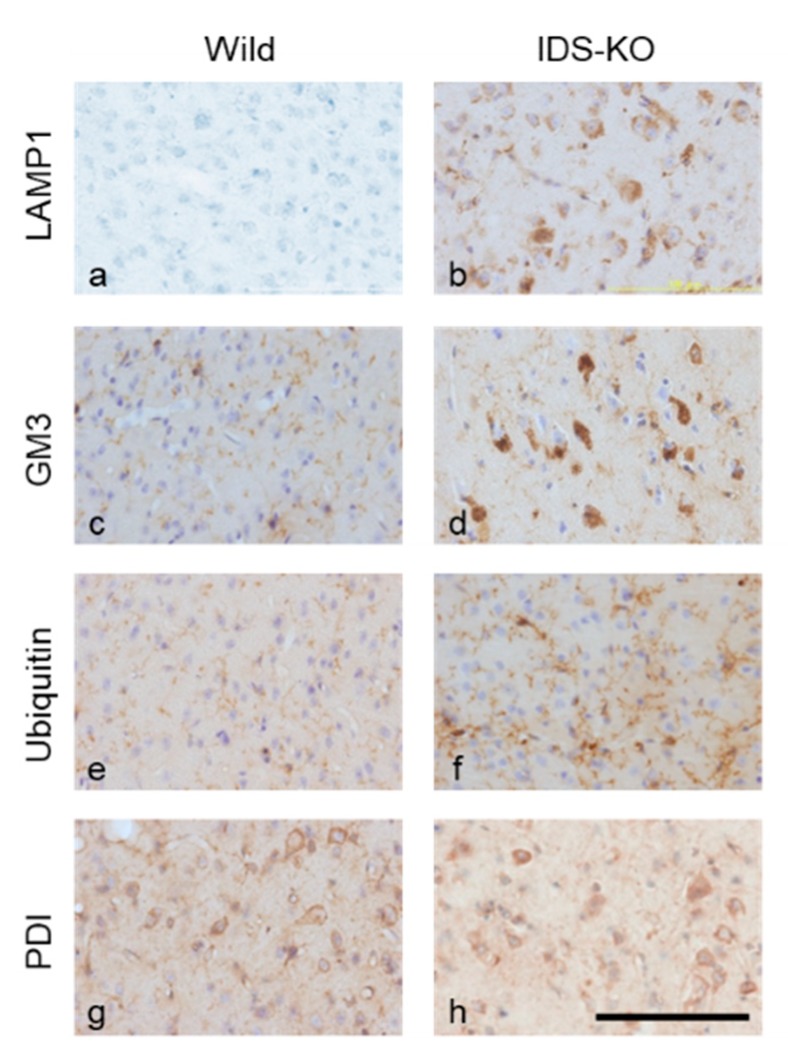
Immunostaining of the cerebral cortex for lysosomal associated protein 1 (LAMP 1; **a**,**b**), GM3 (**c**,**d**), ubiquitin (**e**,**f**), and protein disulfide isomerase (PDI; **g**,**h**). Panels a, c, e, and g were obtained from wild mice, and b, d, f, and h were from IDS-KO mice. A number of cells in the IDS-KO mouse brain were immunopositive for LAMP1, GM3, and ubiquitin; this was not observed in wild-type mice. Both groups had PDI-positive cells, but there was no significant difference in the number. Bar = 100 μm.

Changes in autophagy-related proteins were examined in IDS-KO and wild-type mice at six months of age. The number of cells in the cerebrum positive for lysosomal associated protein 1 (LAMP 1) was higher in IDS-KO than in wild-type mice (Figure 5a,b). Similarly, IDS-KO mice had more cells immunopositive for GM3 ganglioside (Figure 5c,d). The microglia were strongly positive for ubiquitin (Figure 5e,f), suggesting that the ubiquitin–proteasome system was enhanced. Protein disulfide isomerase (PDI) immunostaining did not differ between the KO and wild-type mice, suggesting no substantial enhancement of the ER stress response (Figure 5g,h).

### 2.5. Microstructural Changes Observed by Electron Microscopy

**Figure 6:**
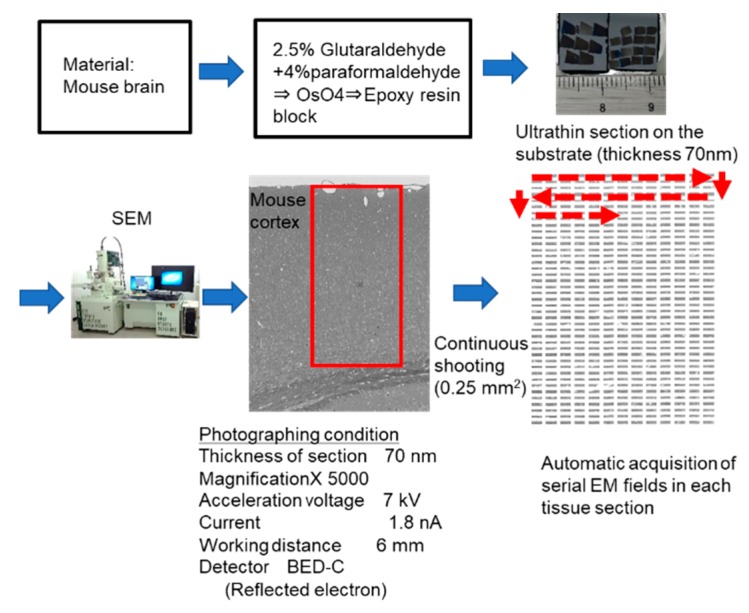
Automatic system for acquiring large-scale scanning electron microscopy images. Epoxy blocks of mouse cerebral cortex are sectioned at 70 nm, mounted on a substrate, and subjected to electron microscopy. Approximately 600 fields of view (0.25 mm^2^ total) are automatically and continuously taken at 5000× with a field emission-scanning electron microscopy (FE-SEM), and one unified image is produced by image processing. Red rectangle including the cortical surface and white matter indicates the automatic acquisition area. Red broken lines and arrows indicate the direction and sequential order of automatic acquisition of electron microscopic images.

We used the automatic acquisition system of sequential electron microscopic (EM) images developed in our laboratory (Figure 6) to count the numbers of cells with autophagy-like vacuoles in wider tissue fields. A large, ultrathin (70 nm) section was prepared in the same manner as for transmission EM and attached to a substrate. Sequential photographs were continuously taken at 5000× magnification with an automatic focus using a field emission-scanning electron microscope (FE-SEM). This system was able to acquire tissue ultrastructural images with a resolution almost equivalent to that of transmission EM. Furthermore, the features of the FE-SEM enabled us to automatically and continuously image about 1000 ultrastructural images across a wide area of tissue (several mm^2^).

### 2.6. Inhibition of Autophagy in Neurons by Using Chloroquine

**Figure 7:**
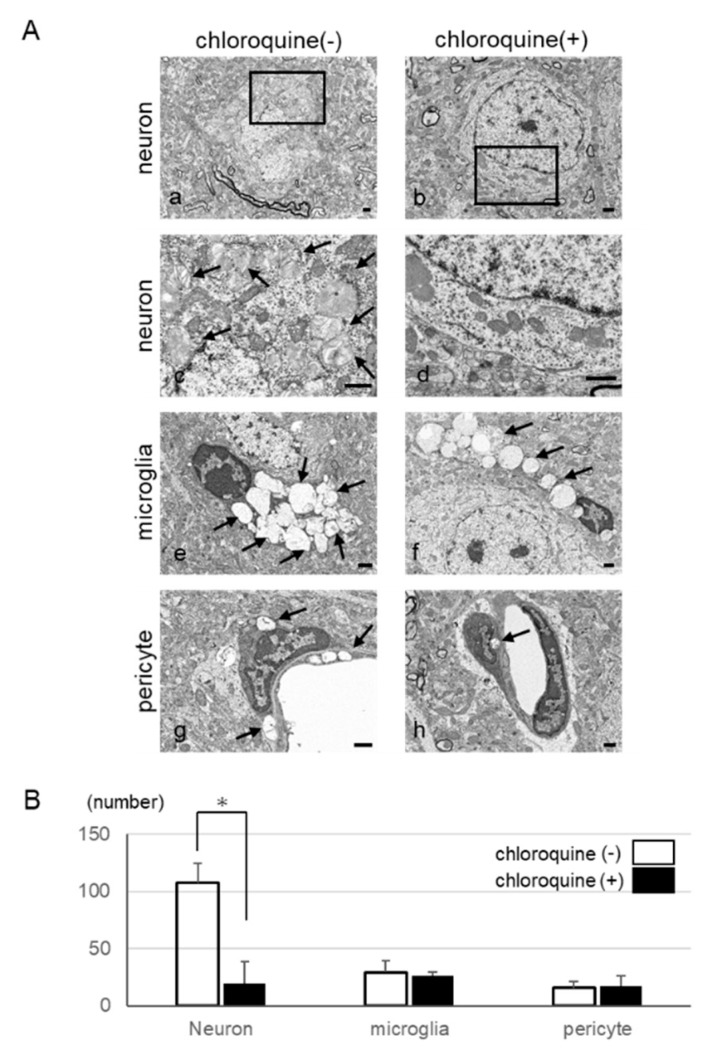
Therapeutic effect of orally administered chloroquine in the cerebral cortex. (**A**) Electron micrographs of the cerebral cortex with or without chloroquine treatment: Representative images of neurons (**a**,**c**), microglia (**e**), and pericytes (**g**) in the cerebral cortex of untreated mice. Representative images of neurons (**b**,**d**), microglia (**f**), and pericytes (**h**) in the cerebral cortex of treated mice. Fewer cytoplasmic vacuoles (arrows) were seen in the neurons after chloroquine treatment than in the untreated group. Panels c and d show magnified views of areas indicated by black rectangles in a and b, respectively. Bar = 1 μm. (**B**) Changes in the number of cells with vacuoles in the cytoplasm with and without the oral administration of chloroquine. The number of neurons showing vacuolation was markedly decreased after the oral administration of chloroquine. However, the number of microglia and pericytes did not change. * *p* < 0.05.

To investigate whether chloroquine, which inhibits autophagy, would preserve cell integrity, we used the automatic acquisition feature of our large-scale SEM system (Figure 6) and examined whether chloroquine treatment led to a decrease in the number of autophagy-like vacuoles. We administered chloroquine orally to IDS-KO mice from 4 to 25 weeks of age. At 25 weeks, the number of neurons with abnormal inclusion bodies or vacuoles was significantly lower in treated than in untreated animals (Figure 7A,B). In contrast, vacuoles remained in the microglia and pericytes and were not markedly different from those in the untreated group (Figure 7A,B). On the basis of these findings, we hypothesize that suppression of autophagy could inhibit the degeneration of neurons and delay the progression of disease.

## 3. Discussion

Autophagy is important for maintaining resting cells such as neurons, for example, by removing misfolded proteins, and the knockout of autophagy-associated genes such as *Atg5* and *Atg7* leads to neurodegeneration in mice [21,22]. The accumulation of substances in lysosomes is thought to damage the autophagy system, leading to its failure and neuronal cell death. Suppression of autophagy increases the effects of treatment for myopathy in a mouse model of Pompe disease [11]. Furthermore, autophagy is activated in a mouse model of GM1 gangliosidosis showing neurodegenerative signs [13]. In Alzheimer’s disease, the abnormal substances that accumulate in the cytoplasm with autophagy cannot be treated and cause cell damage. In contrast, in LSD, non-metabolites accumulate inside the lysosomes and abnormal autophagy causes cell damage. In LSD, it is unclear whether suppression or promotion of autophagy is better for deterring neuronal cell death. Furthermore, the mechanisms underlying the disorders in LSDs may differ depending on the substances that accumulate. In GM2-gangliosidosis, a large amount of GM2-ganglioside accumulates in the brains of model mice, causing neuropathy. In contrast, in a mouse model of MPS IIIB (Sanfilippo disease), heparin sulfate—supposedly the main substance to accumulate in MPS IIB—does not accumulate to a large extent [23]; rather, the secondary accumulation of GM3 ganglioside causes abnormal phosphorylation of tau protein and CNS symptoms [24]. In addition, ganglioside is thought to signal the induction of apoptosis by ER stress [25]. In 1997, Elleder et al. observed the accumulation of SCMAS in the neurons of patients with neuronal ceroid lipofuscinoses [18]. In addition, they reported that SCMAS accumulated in the cerebral neurons of patients with MPS I, II, and IIIA; Niemann–Pick diseases; and GM1 and GM2 gangliosidoses [18], suggesting that the neuropathy has a common mechanism in these LSDs. Ryazantsev et al. subsequently reported the accumulation of SCMAS in the cerebral neurons of a mouse model of MPS IIIB [26], and we hypothesized that the accumulation of SCMAS depends on the level of autophagy, such that SCMAS could be used as a marker of autophagy. At three months after birth, SCMAS-positive cells were predominantly seen in the microglia and pericytes, with few found in neurons, but at 25 weeks, the number of SCMAS-immunopositive cells in neurons was greater than the number in microglia. These results suggest that initially, the autophagy reaction mainly occurs in the microglia and that autophagy of the neurons may occur after the microglia are damaged. We hypothesized that the layered and fingerprint structures, spiral structures, and vacuoles of the neurons in the IDS-KO mice were products of mitochondrial autophagy. The mitochondria of degenerated neurons were immunopositive for SCMAS antibodies, suggesting that the presence of numerous cytoplasmic vacuoles may reflect mitochondrial degeneration, and SCMAS antibodies are detected when mitochondria are destroyed by autophagy [24]. In fact, SCMAS and p62 antibodies, which are localized in protein aggregates and abnormal mitochondria, show positive immunostaining in IDS-KO mouse neurons [18,19]. The abnormally high degree of autophagy of mitochondria in the neurons of the IDS-KO mice may be the main cause of their disability. We found that early continuous administration of chloroquine—an autophagy-suppressing drug—inhibited the degeneration of neurons in MPS II model mice. We had planned to investigate the effects of intraperitoneal administration of rapamycin (an inducer of autophagy) from four weeks of age; however, side effects of the drug severely weakened the mice, and the experiment was stopped at 14 weeks of age. Similarly, when another autophagy-promoting drug (verapamil) was administered, the condition of the animals worsened, and they died. These results suggest that autophagy is abnormally enhanced in MPS II, and that suppression of autophagy suppresses the degeneration of neurons, leading to a therapeutic effect. The enhanced levels of p62/SCMAS reflect impaired autophagy and may be the result of increased initiation of autophagy and/or autophagosome-lysosome fusion, or a reduced ability of lysosomes to degrade the contents delivered to the lysosomes. Previous studies have suggested that chloroquine inhibits autophagosome fusion to lysosomes [27]. Therefore, it is expected to increase autophagosomes before the fusion. In this study, the number of small structures surrounded by the membrane seemed to be increased in neurons of IDS-KO mice treated with chloroquine in EM images. Many of these structures were surrounded by a single membrane, and no obvious double-confined membrane structures were seen. We speculate that those were autophagosomes. It is reported that autophagic vacuoles in the early stages are characterized by a double membrane structure, but late ones are often recognized as single membrane vacuoles [28]. On the other hand, it was reported that chloroquine also has possible effects such as enhancing exocytosis and lysosomal enzyme activity [29,30,31]. In the viewpoint of those effects, we could not observe any finding in our EM images. We hypothesized that the accumulation of substances in the lysosomes causes damage, as well as failure of the autophagy system, result in neuronal cell death. Inhibition of autophagy had a therapeutic effect in a mouse model of Pompe disease myopathy [11]. In terms of the side effects of chloroquine, retinitis pigmentosa and retinal atrophy are serious problems in clinical use. Other side effects reported include ototoxicity; heart involvements; slowly progressive muscle weakness; and mental changes such as aggression, personality change, and memory loss [32]. Therefore, we need to search for autophagy-regulating drugs that are much safer than chloroquine. Furthermore, activation of autophagy has been reported in GM1 gangliosidosis model mice showing neurodegenerative signs [13]. Treatments for LSDs include ERT [33] and bone marrow transplantation [34], and gene therapy is being investigated [35,36]. However, ERT is not effective against neurodegenerative signs, which occur in 70% of patients, because the enzymes do not cross the blood–brain barrier. Furthermore, nerve tissue impairment represents an irreversible change, so that early intervention is required to prevent permanent damage. With regard to stem cell transplantation, the invasiveness and the immune response to transplanted cells are problems to be solved [37,38,39,40]. Chaperone therapy with low-molecular-weight substances that allow passage through the blood–brain barrier, as well as therapy with substrate production inhibitors, have been studied [41,42]. Some drugs administered orally to inhibit substrate synthesis have already been applied clinically; however, the effects are insufficient. In this study, autophagy suppression was observed to protect neurons in the CNS of a mouse model of MPS II. Suppression of autophagy could thus be a novel therapeutic strategy for treating the CNS manifestations of LSDs, for which other treatments have had little effect. Because rapamycin accelerate autophagy by inhibiting mammalian target of rapamycin (mTOR) activity, enhancing agents of mTOR may be potential therapeutic drugs for MPS [43,44].

### 3.1. Limitations

We were not able to investigate the effect of rapamycin, an autophagy inducer, on the immunohistological markers in the mouse brain because of side effects. In this study, behavioral outcomes were not evaluated because the mice did not show any apparent deficits within the first nine months of age. Chloroquine cannot be recommended for clinical use given its potential side effects, but we selected it for this study in IDS-KO mice to evaluate the mechanism of autophagy through an examination of the morphological effects.

### 3.2. Conclusions

In MPS II model mice, enhanced levels of p62 and SCMAS were observed. This is suggestive of impaired autophagy throughout the brain and progresses over time. Vacuoles suggestive of autophagy are found in neurons, microglia, and pericytes. However, administration of chloroquine, which has an autophagy-suppressing effect, reduced the vacuole-like changes of neurons, and a protective effect was observed. These results suggest a new therapeutic approach to treating the effects of LSDs on the nervous system; such treatments have been limited by the blood–brain barrier.

## 4. Methods

### 4.1. Animal Model

The male MPS II model mice (IDS-KO mice) were produced at Japan Chemical Research Pharmaceuticals Co., Ltd. (JCR, Hyogo, Japan) [45]. These model mice were derived from the C57BL/6 strain and were produced by deleting exons 2 to 5 of the IDS gene. C57BL/6 wild-type mice were used as controls. All of the experimental protocols were approved by the Ethics Review Committee for Animal Experimentation of Osaka City University Graduate School of Medicine (No.421 and No.11004: 10th August, 2011). The mice were kept in a controlled environment (temperature, 22 to 23 °C; humidity, 50% to 60%) under a 12-h light/dark cycle with ad libitum access to water and food.

### 4.2. Immunohistochemistry and Light Microscopy

The time course of autophagy and the type and brain sites of cells affected were examined in the brain tissue of wild-type and IDS-KO mice. We mainly visualized phosphorylated p62 (the active type of p62 associated with polyubiquitinated proteins to autophagosomes) and SCMAS, which appears when mitochondria undergo autophagy. At nine weeks and three, six, and nine months after birth (*n* = 3 per age group), the mice were sacrificed and their brains were collected. For the collection of brain tissue, age-matched wild-type and IDS-KO mice were deeply anesthetized with chloral hydrate (75 mg/kg, i.p.) and perfused with saline from the left ventricle. The mice were then perfused with 2% paraformaldehyde and 1.2% picric acid in 0.1 M phosphate for light microscopy. After perfusion fixation, the brain was removed and frozen with dry ice powder, after which 16 μm coronal sections were cut with a cryostat (Microm HM 560, Microedge Instrument, Inc., Surrey, BC, Canada) and thaw-mounted onto 3-aminopropyltriethoxysilan-coated slides. For histopathological analysis, HE and TB staining was performed on some sections from each animal. The avidin-biotin complex method (ABC method) was used for immunohistochemistry. The sections were pretreated with 0.3% H_2_O_2_ in phosphate-buffered saline (PBS) and incubated for 30 min at room temperature (RT) with PBS containing 10% normal goat serum. These sections were then incubated with an anti-SCMAS rabbit monoclonal antibody (1:500, Abcam, Cambridge, UK), anti-p62 (1:100, MBL, Nagoya, Japan), anti-LAMP1 rabbit polyclonal antibody (1:300; Santa Cruz Biotechnology, Santa Cruz, CA, USA), anti-GM3 mouse monoclonal antibody (1:50, Cosmo Bio, Tokyo, Japan), anti-ubiquitin mouse monoclonal antibody (1:500, NOVUS, Littleton, CO, USA), or anti-PDI mouse monoclonal antibody (1:200, ENZO Life Science, New York, NY, USA) overnight at 4 °C. After washes with PBS, the sections were incubated with goat biotinylated anti-rabbit IgG (1:500; Vector Labs, Burlingame, CA, USA) or goat biotinylated anti-mouse IgG (1:500; Vector Labs) for 30 min at RT and then incubated in avidin–biotin horseradish peroxidase complex (Vector Labs, Inc.) for 60 min at RT. The sections were stained in Tris–HCl containing 3,3′-diaminobenzidine (0.2 mg/mL) and 0.003% H_2_O_2_ and counterstained with hematoxylin. For the double-labeling study, the sections were stained with anti-SCMAS rabbit monoclonal antibody (1:100) in combination with an anti-p62 rat monoclonal antibody (1:200) or anti-NeuN mouse monoclonal antibody (1:100, Millipore, Burlington, MA, USA). Sections were also stained with anti-p62 rat monoclonal antibody (1:200) in combination with anti-NeuN mouse monoclonal antibody (1:100, Millipore), anti-iba-1 rabbit polyclonal antibody (1:400, Wako, Richmond, VA, USA), or anti-PDGFR-β rabbit polyclonal antibody (1:100, Santa Cruz) overnight at 4 °C. After three washes with 0.3% Triton X-100 in PBS (TBS-T), sections were incubated at RT for 3 h with appropriate secondary antibodies conjugated with either Cy2 or Cy3 (1:200, Jackson Immuno Research, West Grove, PA, USA). After three washes with TBS-T, the sections were mounted with Hoechst solution (1:1000, Dojindo Laboratories, Tokyo, Japan) and observed under a confocal laser microscope (Digital Eclipse CI; Nikon, Tokyo, Japan).

### 4.3. Electron Microscopy

At six months after birth, IDS-KO and wild-type mice (*n* = 3 per group) were perfused and fixed with a 2% glutaraldehyde (GA)–4% paraformaldehyde (PFA) mixture under general anesthesia. The brains were removed and further fixed with 4% paraformaldehyde at 4 °C overnight. Coronal slices (100 μm thick) were prepared, post-fixed in osmium, dehydrated, and embedded in epoxy resin. Ultrathin (70 nm) cortical sections were cut, mounted on pieces of silicon wafers, and contrasted with uranyl acetate and lead citrate.

Scanning electron microscopic images of the brain specimens were obtained using a backscattered electron detector (BED-C; voltage, 7 kV; PC current, 1.8 nA; work distance, 6) in a JSM-7800F SEM (JEOL, Tokyo, Japan).

### 4.4. Immunoelectron Microscopy for SCMAS

An immunoelectron microscopic study was performed to observe SCMAS that appeared when mitochondria underwent autophagy. In the study, six-month-old wild-type and IDS-KO mice (*n* = 3 each) were transcardially perfused with 0.1% GA and 4% PFA under general anesthesia, and their brains were immersed in the same fixative at 4 °C for 16 h and cut into 50 µm thick sections with a microslicer (Leica, VT1000S, Wetzlar, Germany). After incubation with anti-SCMAS antibody (1:500, Abcam, Cambridge, UK), the sections were subjected to the ABC method as described for light microscopy. After the diaminobenzidine (DAB) reaction, the sections were post-fixed with 1% OsO_4_ for 1 h, dehydrated with alcohol, and flat-embedded on siliconized glass slides in epoxy resin. Ultrathin cortex sections were cut, mounted on silicon wafers, and contrasted with 2% uranyl acetate and lead citrate, after which electron micrographs were obtained using a BED-C in a JSM-7800F (JEOL Japan).

### 4.5. Administration of Chloroquine to IDS-KO Mice

To investigate the effect of suppressing autophagy on neuronal damage, chloroquine (10 mg/day) was orally administered to IDS-KO mice (*n* = 6) for 25 weeks from 28 days (4 weeks) after birth. IDS-KO mice (*n* = 6) not given chloroquine were used as controls. After the end of the administration, the mouse brains were fixed and prepared for imaging with conventional EM, and the number of neurons having vacuoles within the cerebral cortex was counted and graphed.

### 4.6. Cell Counting by Automatic Acquisition System of Sequential EM Images in Wider Tissue Fields and Statistical Analysis

We used the automatic acquisition system of sequential EM images to count autophagy-like cells in wider fields of the brain tissues. We used JEOL’s JSM-7800F SEM, with BED-C at a voltage of 7 kV, a current of 1.8 nA, and a work distance of 6 mm.
